# Analysis of PI3K Pathway Associated Molecules Reveals Dysregulated Innate and Adaptive Functions of B Cells in Early Diffuse Cutaneous Systemic Sclerosis

**DOI:** 10.3390/ijms22062877

**Published:** 2021-03-12

**Authors:** Diána Simon, Szabina Erdő-Bonyár, Judit Rapp, Péter Balogh, Tünde Minier, Gabriella Nagy, László Czirják, Tímea Berki

**Affiliations:** 1 Clinical Center, Department of Immunology and Biotechnology, University of Pécs Medical School, H-7624 Pécs, Hungary; erdo-bonyar.szabina@pte.hu (S.E.-B.); rapp.judit@pte.hu (J.R.); balogh.peter@pte.hu (P.B.); berki.timea@pte.hu (T.B.); 2 Clinical Center, Department of Rheumatology and Immunology, University of Pécs Medical School, H-7632 Pécs, Hungary; minier.tunde@pte.hu (T.M.); nagy.gabriella@pte.hu (G.N.); czirjak.laszlo@pte.hu (L.C.)

**Keywords:** B cells, systemic sclerosis, dcSSc, PI3K pathway, CD180, SPP1, NF-κB, IL-10, switched memory B cells

## Abstract

B cell activation is an early event in the development of systemic sclerosis (SSc). The classical activation of B cells downstream of the B-cell receptor (BCR) involves the phosphatidylinositol-3 kinase (PI3K) pathway that integrates the effects of multiple co-stimulatory receptors. Our analysis of PI3K pathway associated molecules in peripheral blood B cells of early diffuse cutaneous SSc (dcSSc) patients showed altered mRNA expression of Toll-like receptor (TLR) homolog CD180, TLR4, complement component 3, IL-4 receptor and secreted phosphoprotein 1 (SPP1). Parallel to this, we found elevated basal SPP1 secretion in dcSSc B cells, but, with BCR + IL-4 receptor co-stimulation, we could not induce further secretion. CD180 stimulation alone resulted in NF-κB activation in more B cells than CD180 + BCR co-stimulation both in dcSSc and healthy control (HC), but the co-engagement increased the phosphorylation of NF-κB only in dcSSc B cells. Additionally, in contrast with HC B cells, the lower basal production of IL-10 by dcSSc B cells could not be elevated with CD180 stimulation. Furthermore, activation via CD180 increased the percentage of CD86+ switched memory (CD27+IgD−) B cells in dcSSc compared to HC. Our results suggest that alternative B cell activation and CD180 dysfunction cause imbalance of regulatory mechanisms in dcSSc B cells.

## 1. Introduction

Systemic sclerosis (SSc) is a connective tissue disease with vascular damage and consequent ischemic-reperfusion injury and multiorgan fibrosis [1]. All these phenomena are clearly involved in the pathogenesis of the disease, but their connection remains elusive. Importantly, these events show strong correlation with accompanying severe immune dysregulation, including the production of scleroderma-specific autoantibodies, secretion of pro-inflammatory and pro-fibrotic cytokines by B cells [2,3,4]. Transcriptome profiling has already identified Th1, Th2 and B cell activation in early SSc skin biopsies [5]. In contrast to early diffuse cutaneous SSc (dcSSc), established disease did not show similar prominent immune signature, suggesting that activation of B cells is an early event in SSc development.

The classical activation of B cells downstream of B-cell receptor (BCR) and CD19 co-receptor engagement involves phosphatidylinositol-3 kinase (PI3K) signaling pathway, which also integrates the effects of the co-stimulatory interleukin 4 (IL-4) receptor (IL-4R) [6,7]. Moreover, Toll-like receptor (TLR) and complement receptor signaling seem to also be mediated by PI3K [8].

The involvement of PI3K pathway was found in fibrogenesis in SSc [9,10]. Dermal fibroblasts of SSc patients showed hyperactivity of PI3K signaling [9] and inhibition of this pathway in an SSc mouse model repressed TGFβ1-induced expression of type 1 collagen in lung and dermal fibroblasts [10]. Additionally, in a murine ischemia-reperfusion injury model, the inhibition of PI3K pathway reduced acute tubular necrosis and the infiltration of B cells into the injured kidneys [11]. Consequently, PI3K signaling seems to be associated with the two fundamental phenomena of SSc [1].

In this study, we analyzed the mRNA expression of molecules associated with PI3K signaling pathway in peripheral blood B cells of patients with early dcSSc and compared to healthy control (HC) B cells. We found that the expression of innate signals related TLR4 and complement component 3 (C3) were upregulated, accompanied by decreased expression of the TLR homolog CD180. Furthermore, we demonstrated elevated mRNA levels of secreted phosphoprotein 1 (SPP1) and IL-4R in B cells of dcSSc patients. We explored the production of SPP1 by B cells upon BCR and IL-4R co-engagement and found that the basal SPP1 secretion by dcSSc B cells reached the level of SPP1 produced by co-stimulated HC B cells. We also studied the influence of the TLR homolog CD180 engagement on B cell activation and found less B cells with phosphorylated NF-κB in dcSSc than in HC. Nevertheless, BCR and CD180 co-ligation increased the phosphorylation of NF-κB in B cells in dcSSc, but not in HC. In addition, we measured lower basal regulatory cytokine IL-10 production by dcSSc B cells, which could not be augmented with CD180 stimulation. We also analyzed the activation of memory B cell subsets and found that the percentage of CD86+ switched memory (CD27+IgD−) B cells was higher in dcSSc than in HC after anti-CD180 treatment. Our results reveal dysregulation of PI3K pathway associated molecules in early dcSSc B cells, which may play a role in B cell dysfunction in SSc.

## 2. Results

### 2.1. mRNA Expression of CD180, TLR4, C3, SPP1 and IL-4R Are Dysregulated in dcSSc B Cells

**Figure 1:**
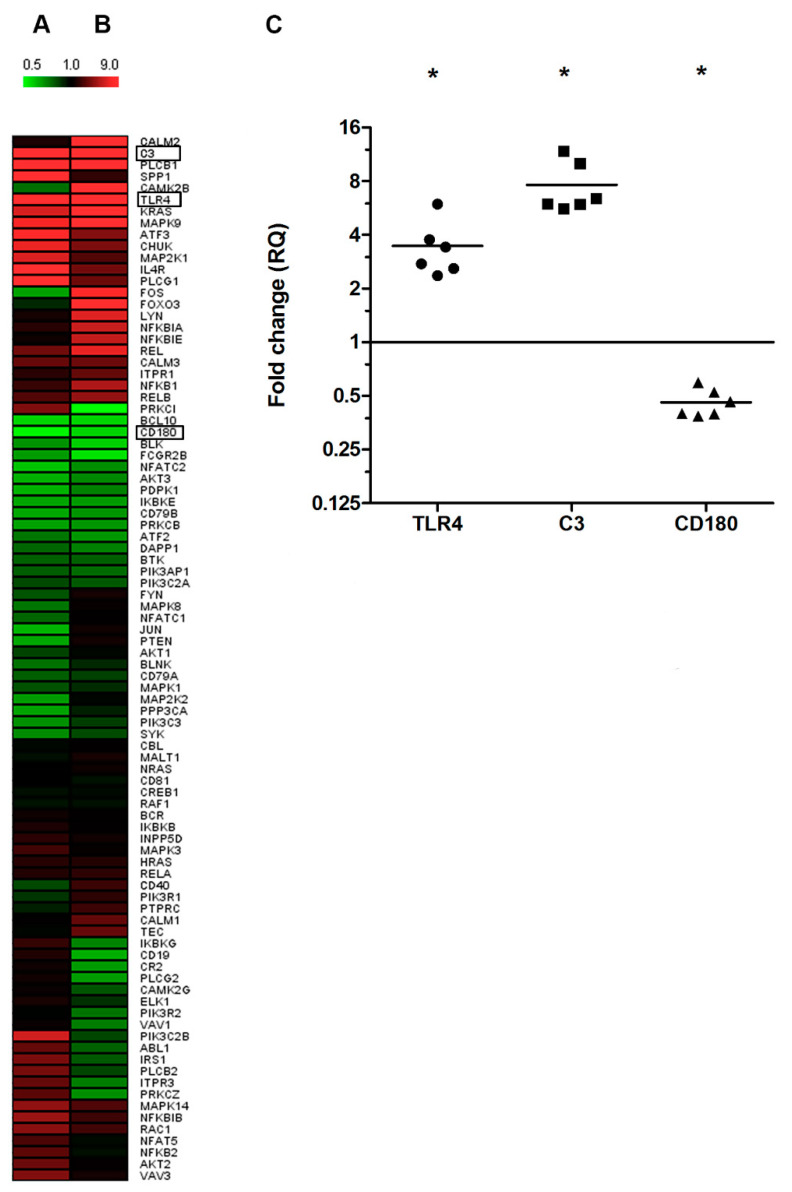
Gene expression analysis of phosphatidylinositol-3 kinase (PI3K) pathway related molecules in B cells. (**A**) Heatmap representing the mRNA expression of 92 molecules associated with PI3K pathway in B cells of early diffuse cutaneous systemic sclerosis (dcSSc) patients untreated (*n* = 5) and (**B**) under immunosuppressive therapy (mycophenolate mofetil, azathioprine or methotrexate, *n* = 3). Gene expression was normalized to healthy controls (HCs) (*n* = 5) and value 1 represents the expression of control samples. Colors represent the level of gene expression where the highly upregulated is bright red and the highly suppressed is bright green. (**C**) Individual qPCR validation of complement component 3 (C3), Toll-like receptor 4 (TLR4) and CD180 gene expression in B cells of dcSSc patients (*n* = 6) compared to HCs (*n* = 5). Gene expression was normalized to HC, and the horizontal line (value 1) represents the expression of control samples. Changes in gene expression are shown as RQ values. Data are presented as means ± SEM. * *p* < 0.05.

First, we determined the mRNA expression of 92 PI3K signaling pathway related genes in B cells of early, untreated dcSSc patients using pooled cDNAs (*n* = 5) and compared to five HCs. We found highly upregulated expression of mRNA for C3 (RQ = 9.333), SPP1 (RQ = 5.305), TLR4 (RQ = 2.654) and IL-4R (RQ = 2.065) in B cells of dcSSc patients. In addition to these secreted (C3 and SPP1) and membrane bound (TLR4 and IL-4R) proteins, we found highly upregulated expression of mRNA of phospholipase-Cβ1 (PLCB1; RQ = 4.698) and phospholipase-Cγ1 (PLCG1; RQ = 2.141), two intracellular enzymes of the PI3K pathway (Figure 1A). Furthermore, we found that the mRNA expression of CD180 was the mostly downregulated (RQ = 0.517) in dcSSc samples compared to HC samples (Figure 1A).

Next, we tested whether immunosuppressive therapy influences the PI3K signaling pathway related mRNA expression in B cells of early dcSSc patients. Using pooled cDNA samples (*n* = 3) we found that the therapy did not cause significant changes in the upregulated mRNA expression of TLR4 (RQ = 4.076), C3 (RQ = 5.572) and PLCB1 (RQ = 6.812). Interestingly, the SPP1, IL-4R and PLCG1 mRNA levels in dcSSc came close to the mRNA levels of HC B cells (RQ = 1.147, RQ = 1.308 and RQ = 1.296. respectively) (Figure 1B). Downregulation of CD180 expression of B cells remained in patients receiving immunosuppressive therapy (RQ = 0.560 and RQ = 0.587) (Figure 1B).

The mRNA expression of C3, TLR4 and CD180 were not influenced by immunosuppressive therapy; therefore, we chose these molecules for further analysis. We determined the mRNA expression of C3, TLR4 and CD180 of B cells with qPCR in further six early dcSSc patients and five HCs individually. The results were similar to what we found with the qPCR array of the 92 genes (Figure 1C).

We previously reported that the CD180 protein expression of B cells was significantly lower in early dcSSc than in HC [12]. In this study, we sought to investigate the TLR4 and C3 protein expression of dcSSc and HC B cells with flow cytometry, but the statistical analysis of the mean fluorescence intensity (MFI) values of C3 and TLR4 labeling of B cells was not applicable due to the high standard deviations of data. Individual variations in C3 and TLR4 expression might be a possible explanation for this (data not shown).

### 2.2. Basal SPP1 Production of dcSSc B Cells Is Similar to IL-4R and BCR Co-Stimulated HC B Cells

**Figure 2:**
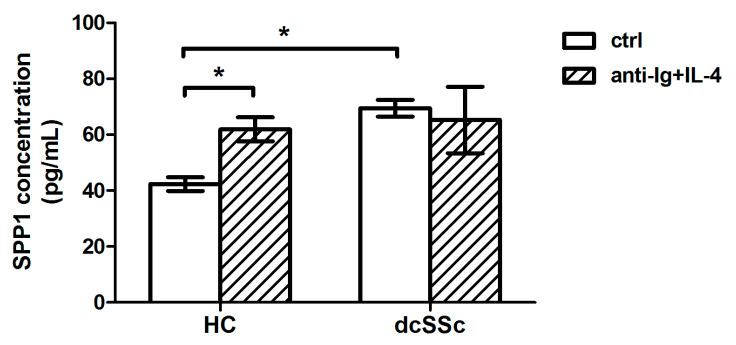
Alternate B cell receptor (BCR) signaling induced secreted phosphoprotein 1 (SPP1) production by B cells. Measurement of SPP1 level in the supernatant of B cells of dcSSc patients (*n* = 4) and HCs (*n* = 3), stimulated with anti-Ig + IL-4 or left unstimulated (ctrl) for 24 h, as measured by ELISA. Data are presented as means ± SEM. * *p* < 0.05.

The combined action of the IL-4R mediated alternate and the classical BCR signaling pathways results in the overexpression of SPP1 [13] and the plasma SPP1 level is elevated in SSc [14]. Moreover, the level of IL-4 is elevated in serum and skin samples of SSc patients [15,16]. Consequently, we analyzed the effect of IL-4R and BCR co-engagement on the production of SPP1 and found that the concentration of SPP1 was significantly higher in the supernatant of unstimulated B cells in dcSSc than in HC. BCR and IL-4R co-stimulation could not further increase the secretion of SPP1 by dcSSc B cells, and the level of SPP1 in the supernatant of co-treated HC B cells only reached the basal SSP1 production of dcSSc B cells (Figure 2).

### 2.3. CD180 Stimulation Alone and in Combination with BCR Ligation Induces the Phosphorylation of NF-κB in dcSSc B Cells

**Figure 3:**
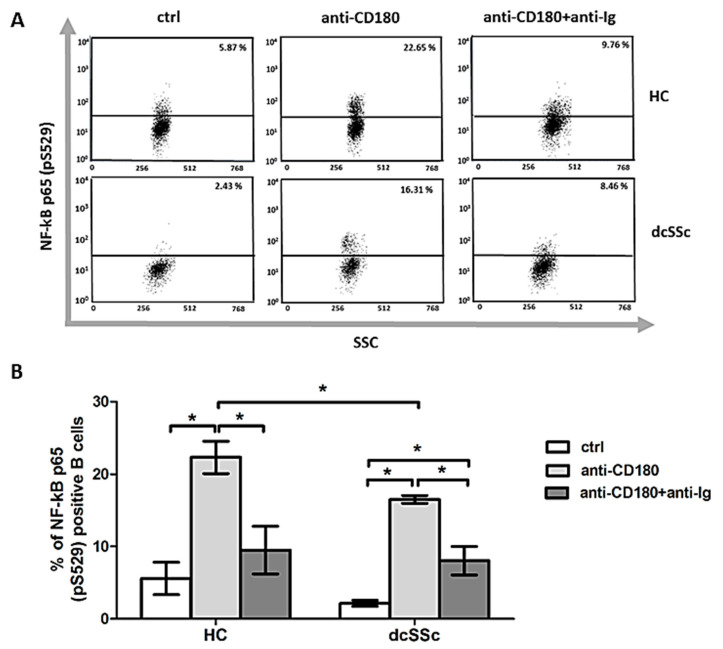
Induction of phosphorylation of NF-κB by CD180 ligation and BCR and CD180 co-engagement. Representative dot plots (**A**) and changes (**B**) of the phosphorylation of NF-κB p65 (pS529) molecule in purified B cells of dcSSc patients (*n* = 5) and in HCs (*n* = 4) after stimulation with anti-CD180 or anti-CD180 + anti-Ig or left unstimulated (ctrl) for 30 min detected by flow cytometry. Data are presented as means ± SEM. * *p* < 0.05.

Ligation of CD180 in chronic lymphocytic leukemia B cells leads to the activation of BTK/PI3K/AKT or p38 MAPK pathways [17] and both pathways have been shown to affect the activity of NF-κB [18,19]. Hence, we investigated the effect of co-stimulation via BCR and CD180 on NF-κB activation and found that CD180 ligation on its own significantly increased the percentage of NF-κB p65 (pS529) positive B cells in both dcSSc and HC compared to unstimulated controls and the phosphorylation of NF-κB in B cells was significantly lower in dcSSc than in HC. However, BCR and CD180 co-engagement significantly enhanced the phosphorylation of NF-κB in dcSSc (Figure 3). Lipopolysaccharide (LPS) is a well-known ligand of TLR4, but TLR4 has also been shown to be activated by endogenous ligands. The expression of both TLR4 and its endogenous ligands are increased in skin and lung biopsy specimens of SSc patients. These endogenous ligands of TLR4 include damage-associated molecules such as connective tissue molecules (e.g., tenascin C) and cellular stress proteins (e.g., HMGB1). Induction of TLR4 signaling leads to pro-fibrotic responses, mediated by MyD88 resulting in the activation of NF-κB [20]. Hence, we tried to trigger TLR4 signaling in HC and dcSSc B cells using LPS, HMGB1 and tenascin C, but we could not detect any changes in the phosphorylation level of NF-κB (data not shown).

### 2.4. Basal IL-10 Production of B Cells Is Lower in dcSSc and Cannot Be Increased with Ligation of CD180

**Figure 4:**
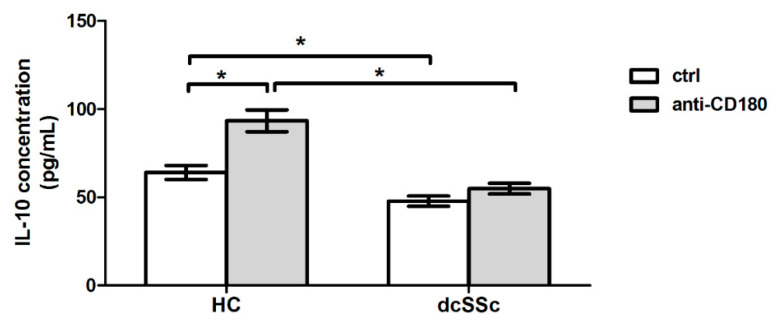
IL-10 production of anti-CD180 antibody stimulated B cells. IL-10 secretion of B cells stimulated with anti-CD180 or left unstimulated (ctrl) for 24 h (dcSSc *n* = 5, HC *n* = 3), as measured by ELISA. Data are presented as means ± SEM. * *p* < 0.05.

Since the function of regulatory B cells in SSc is impaired [21], and we previously found that the CD180 expression of these cells is downregulated by anti-CD180 treatment [12], we investigated whether stimulation of B cells via CD180 has different effect on IL-10 production in dcSSc and HC. The concentration of IL-10 in the supernatant of both unstimulated and anti-CD180 stimulated B cells were significantly lower in SSc than in HC. Moreover, ligation of CD180 significantly increased the IL-10 production of B cells only in HC (Figure 4).

### 2.5. CD180 Expression Is Decreased in Naive and Double Negative B Cells in dcSSc

**Figure 5:**
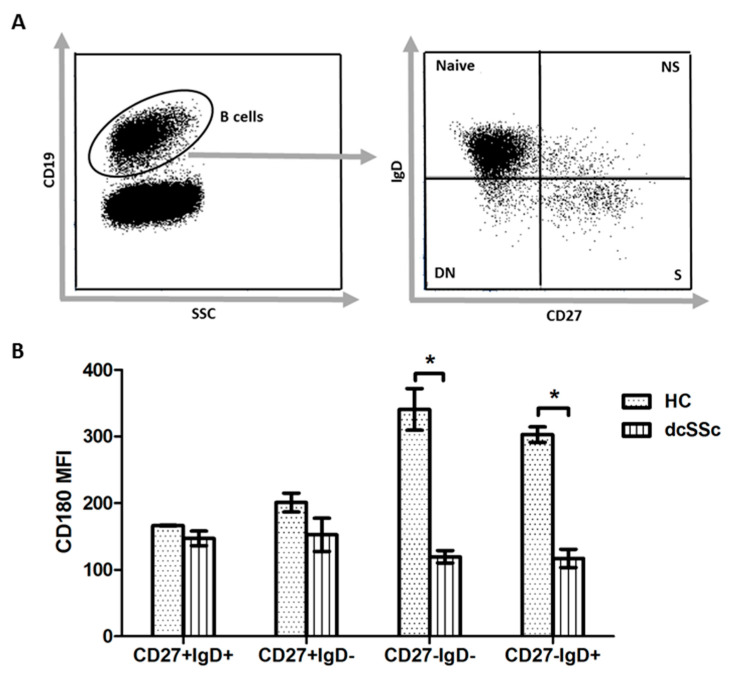
Expression of CD180 in B cell subsets. (**A**) Gating strategy of CD19+ B cells stained with CD27 and IgD to define the following four subsets: CD27+IgD+ non-switched memory (NS) B cells, CD27+IgD− switched memory (S) B cells, CD27−IgD− double negative (DN) B cells and CD27−IgD+ naive B cells. (**B**) The mean fluorescence intensity (MFI) of CD180 in NS, S, DN and naive B cells in dcSSc (*n* = 4) and HC (*n* = 3), as measured by flow cytometry. Data are presented as means ± SEM. * *p* < 0.05.

As we found that the expression of CD180 was lower in total B cells in dcSSc than in HC, we compared the level (mean fluorescence intensity, MFI) of CD180 in B cell subsets between dcSSc and HC. We analyzed the following B cell subsets defined by CD27 and IgD staining: CD27+IgD+ non-switched memory (NS), CD27+IgD− switched memory (S), CD27−IgD− double negative (DN) and CD27−IgD+ naive B cells. We found that the MFI of CD180 labeling was lower in naive and DN B cells in dcSSc than HC (Figure 5).

### 2.6. Stimulation Via CD180 Enhances the Activation of Switched Memory B Cells in dcSSc Compared to HC

**Figure 6:**
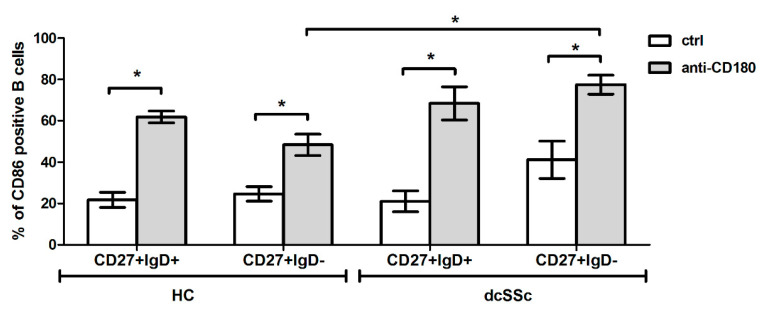
The percentage of CD86 positive B cells in CD27+IgD+ non-switched memory and CD27+IgD− switched memory B cell subsets in anti-CD180 antibody stimulated and unstimulated (24 h) dcSSc (*n* = 4) and HC (*n* = 5) B cells, as measured by flow cytometry. Data are presented as means ± SEM. * *p* < 0.05.

Activated memory B cells are reported in SSc by multiple research groups including ours [22,23,24,25]. According to our previous results memory B cells are activated via CD180 [26], thus we compared the CD86 expression of non-switched (CD27+IgD+) and switched (CD27+IgD−) memory B cell subsets in dcSSc and HC after anti-CD180 stimulation and found that the percentage of CD86+ switched memory B cells was significantly higher in dcSSc than in HC. Nevertheless, the frequency of CD86+ cells was increased in both memory B cell subsets both in dcSSc and HC (Figure 6).

## 3. Discussion

The involvement of PI3K pathway in fibrosis and ischemia/reperfusion injury—two key mechanisms in the pathogenesis of SSc—has already been shown [9,10,11]. PI3K pathway also plays an important role in B cell differentiation, activation, survival and migration [6] and the activation of B cells is an early event in the pathogenesis of SSc. We were the first to investigate the expression of 92 PI3K signaling pathway related genes in peripheral blood B cells of early dcSSc patients and found highly upregulated mRNA expression of C3, TLR4, SPP1 and IL-4R accompanied by decreased CD180 mRNA level.

SPP1 has pro-inflammatory and pro-fibrotic properties and also regulates fibroblast activation and myofibroblast differentiation [27,28]. The Th2 cytokine IL-4 also contributes to inflammation and fibrosis in SSc [29] and inhibition of IL-4 or its receptor has already been suggested as potential therapy in SSc [30]. Guo et al. reported that the combined action of the IL-4R mediated alternate and the classical BCR signaling pathways resulted in the overexpression of SPP1 [13], which is in agreement with our finding of increased production of SPP1 of HC B cells after IL-4R and BCR co-engagement. We also found that the SPP1 concentration in the supernatant of unstimulated B cells was higher in dcSSc than in HC, which is supported by the elevated plasma SPP1 level described in SSc [14]. As Guo et al. [13] used naive B cells for the experiment, the increase in naive B cells in SSc compared to HC might explain the mRNA overexpression of SPP1 and IL-4R in dcSSc, as well as the differences in the basal production of SPP1 in dcSSc and HC. However, BCR and IL-4R co-stimulation could not further elevate the SPP1 production of dcSSc B cells, and this co-stimulation increased the SPP1 production of HC B cells reaching the level of unstimulated dcSSc B cells. Our results suggest the role of the alternative B cell activation in the pathogenesis of SSc.

B cells have mainly been investigated regarding their adaptive functions in SSc, thus it was unexpected to find the elevated expression of innate immune molecules TLR4 and C3 in peripheral blood B cells of dcSSc patients. TLRs recognize pathogen and damage associated molecular patterns and have been identified as key drivers of fibrogenesis in SSc, dominated by TLR4. Expression of TLR4 and its endogenous ligands is elevated in skin biopsies of SSc, and dermal and lung fibrosis was attenuated in bleomycin-treated TLR knockout mice [20]. Blockade of TLR4-dependent signaling was already suggested as therapy in SSc [1]. Our investigation on TLR4 revealed that neither LPS nor its endogenous damage associated ligands (HMGB1 and tenascin C) were able to activate signaling through TLR4. Local complement activation and abnormal deposition of complement components were detected in skin biopsies of SSc patients, but not in HCs [26]. According to its originally described function, C3 acts exclusively extracellular, but new evidence has shed light on its intracellular functions. Furthermore, the latter are thought to be evolutionary more ancient [31]. Intracellular C3 expression has been linked to cell survival and metabolism in T cells [32]. Thus, our finding that C3 gene expression is upregulated in dcSSc B cells may point to a possible role of C3 in B cell dysfunctions in dcSSc.

CD180 was originally defined as a B cell surface molecule mediating activation and proliferation [33] and was later described as a TLR homolog expressed by dendritic cells and macrophages. We previously demonstrated that the expression of CD180 in anti-CD180 activated B cells was decreased [12], supporting the theory proposed by Chaplin at al. [34] that CD180 can be internalized after ligation, thus decreased level of CD180 could reflect the enhanced activation of B cells via CD180. The MyD88-independent pathways of CD180 signaling under pathologic conditions has only been investigated in chronic lymphocytic leukemia cells showing the activation of BTK/PI3K/AKT or p38 MAPK pathways [17]. Both of these pathways have been shown to affect the activity of NF-κB [18,19]. According to our results, the co-activation via CD180 and BCR and anti-CD180 stimulation on its own resulted in increased phosphorylation of NF-κB in dcSSc B cells, but the level of phosphorylation was not higher in dcSSc than in HC, even though the CD180 MFI of naive B cells was lower in dcSSc than in HC and naive B cells are increased in SSc compared to HC. Since NF-κB is a central mediator of pro-inflammatory gene induction and functions [35], activation of dcSSc B cells via CD180 and BCR could contribute to inflammation, which is a crucial player in the pathogenesis of SSc.

The impaired function of regulatory B cells has already been described in SSc [21], which is supported by our results showing that the basal IL-10 secretion by B cells was lower in dcSSc. Pararasa et al. [36] analyzed the intracellular IL-10 staining in CD27−IgD−, CD27−IgD+, CD27+IgD− and CD27+IgD+ sorted peripheral blood B cells and found that only CD27−IgD+ B cell were capable of IL-10 production. Therefore, the lower basal IL-10 production of B cells in dcSSc than HC cannot be explained by the increased naive and decreased memory B cells in SSc compared to HC. Furthermore, only HC B cells produced higher amounts of IL-10 after anti-CD180 treatment, suggesting the role of CD180 in the dysfunction of IL-10 producing regulatory B cells in dcSSc.

Anti-CD180 stimulation activates over 85% of human and mouse B cells in vitro [37], and we found that it activated the investigated memory B cell subsets both in dcSSc and HC. In our previous study, we reported that the relative frequency of switched memory B cells, responsible for the production of pathologic autoantibodies, is associated with the severe form of SSc [25]. Here, we demonstrated that the percentage of CD86+ switched memory B cells was higher after anti-CD180 treatment in dcSSc than in HC, suggesting that B cell activation via CD180 could have a role in the pathological antibody production in SSc. According to our results, anti-CD180 antibody therapy, which has already been suggested in SLE [38], could enhance B cell dysfunction in SSc.

## 4. Materials and Methods

### 4.1. Patients

Twenty-one patients with early dcSSc (disease duration was 2.05 (±1.2) years based on the date of the first non-Raynaud’s symptom) were enrolled for the study. They all fulfilled the 2013 ACR/EULAR SSc classification criteria. Mean (SD) age at enrollment was 46.43 (±17.2) years, mean (SD) modified Rodnan skin score was 14.65 (±7.6) points, and frequent internal organ involvements were gastrointestinal involvement (57.1%), cardiac involvement (42.9%) and interstitial lung disease (33.3%). The aforementioned internal organ changes corresponded to the involvement seen in early phase of disease. The detailed characteristics of the patients are shown in Table 1. Controls (*n* = 18) were age- and sex-matched healthy individuals (HC). All participants gave a written informed consent to the study, after approval by the Hungarian National Ethics Committee.

### 4.2. Peripheral Blood Mononuclear Cell Isolation and B Cell Separation

Peripheral blood mononuclear cells (PBMCs) were isolated by Ficoll-Paque Plus (GE Healthcare, Chicago, IL, USA) density gradient centrifugation of peripheral blood samples. MACS B cell isolation kit II (Miltenyi Biotech, Bergisch Gladbach, Germany) was used for negative selection of B cells according to the manufacturer’s instructions.

### 4.3. Cell Stimulation

For Phosflow assay, 2 × 10^5^ B cells per condition were seeded onto a 96-well plate in RPMI-1640 (Merck KGaA, Darmstadt, Germany) culture medium without FBS (SSc *n* = 5, HC *n* = 4) for 1 h. B cells were then stimulated with LEAF Purified anti-human CD180 (RP105) antibody (Clone: MHR73-11) (BioLegend, San Diego, CA, USA) at 1 µg/mL (anti-CD180), or anti-CD180 in combination with affinity purified F(ab’)2 fragment of goat anti-human IgM + IgG (H + L) (Jackson Immunoresearch Laboratories, London, UK) at 2.5 µg/mL (anti-Ig) or left unstimulated for 30 min at 37 °C. To investigate the activation of the memory B cell subsets, 5 × 10^5^ PBMCs were stimulated with anti-CD180 or left unstimulated for 24 h 37 °C (SSc *n* = 4 HC *n* = 5). To assess IL-10 production, 2 × 10^5^ B cells were stimulated with anti-CD180 or left unstimulated for 24 h at 37 °C (SSc *n* = 5, HC *n* = 3). To investigate SPP1 secretion, 2 × 10^5^ B cells were co-treated with anti-Ig + IL-4 (Human IL-4 Recombinant Protein at 10 ng/mL) (Thermo Fisher Scientific, Waltham, MA, USA) or left unstimulated for 24 h at 37 °C (SSc *n* = 4, HC *n* = 3).

### 4.4. RNA Isolation, cDNA Synthesis and qPCR

Total RNA was extracted from isolated B cells using NucleoSpin RNA XS kit (Macherey-Nagel Inc, Bethlehem, PA, USA). Following cDNA generation (High Capacity cDNA Reverse Transcription Kit, Thermo Fisher Scientific, Waltham, MA, USA), TaqMan™ Human B cell PI3K Signaling Array (Thermo Fisher Scientific, Waltham, MA, USA) was used to detect the expression of 92 genes using pooled cDNA samples of early untreated dcSSc patients (*n* = 5), patients treated with immunosuppressive therapy (*n* = 3) and HCs (*n* = 5). Amplifications were run on Applied Biosystems 7500 RT-PCR System (Thermo Fisher Scientific, Waltham, MA, USA). Gene expression was analyzed with 7500 Software v2.0.6 (Thermo Fisher Scientific, Waltham, MA, USA) and normalized to 4 housekeeping genes. The mRNA expression of TLR4, CD180 and C3 was also analyzed individually in early dcSSc patients (*n* = 6) and HCs (*n* = 5) using Applied Biosystems TaqMan Gene Expression Assays (Thermo Fisher Scientific, Waltham, MA, USA) and normalized to GAPDH. Fold changes (RQ) were calculated according to the 2-ddCT method.

### 4.5. Cell Phenotyping

To analyze the expression of CD180 on B cells defined by CD27 and IgD staining and investigate the expression of the activation marker CD86 of CD27+IgD+ non-switched and CD27+IgD− switched memory B cell subsets after anti-CD180 stimulation, samples of 4 dcSSc patients and 5 HCs were analyzed using the combination of anti-human CD19-AmCyan (SJ25C1, Becton Dickinson, Franklin Lakes, NJ, USA), anti-human CD27−PE/Cy7 (M-T271, BioLegend, San Diego, CA, USA), anti-human IgD−PerCP (IA6-2, BioLegend, San Diego, CA, USA), anti-human CD180-PE (G28-8, Becton Dickinson, Franklin Lakes, NJ, USA) and anti-human CD86-Pacific Blue (IT2.2, BioLegend, San Diego, CA, USA) following the manufacturer’s protocols. Briefly, PBMCs were incubated with the appropriate antibodies for 30 min on ice, washed twice in phosphate buffered saline and fixed with 1% paraformaldehyde. Fluorescence of the labeled cells was recorded using a FACS Canto II flow cytometer (Becton Dickinson, Franklin Lakes, NJ, USA) and analyzed using FCS Express 6 software (De Novo Software, Pasadena, CA, USA).

### 4.6. Analysis of Phosphorylation of NF-κB p65

For Phosflow assay, we used the PE Mouse anti-human NF-κB p65 (pS529) (K10-895.12.50, Becton Dickinson, Franklin Lakes, NJ, USA) antibody. Phosflow assay was performed in purified B cells of dcSSc patients (*n* = 5) and HCs (*n* = 4) according to BD Phosflow Protocol using BD Cytofix Fixation Buffer and BD Perm II Buffer (BD Biosciences, Franklin Lakes, NJ, USA). Briefly, after stimulation, cells were immediately fixed with pre-warmed Cytofix Fixation buffer for 10 min at 37 °C. Cells were then washed and permeabilized with pre-cooled Perm Buffer II for 30 min on ice. After washing the cells three times, they were stained with PE Mouse anti-human NF-κB p65 (pS529) and incubated for 30 min at room temperature. Then, cells were washed and immediately measured without fixation. Fluorescence of the labeled cells was recorded using a FACS Canto II flow cytometer (Becton Dickinson, Franklin Lakes, NJ, USA) and analyzed using FCS Express 6 software (De Novo Software, Pasadena, CA, USA).

### 4.7. SPP1 ELISA

The supernatant obtained from dcSSc (*n* = 4) and HCs (*n* = 3) B cells co-treated with anti-Ig + IL-4 or left unstimulated for 24 h were collected and stored at −80 °C until being measured. The concentration of SPP1 was determined with Human Osteopontin (OPN) DuoSet ELISA kit (Bio-Techne, Minneapolis, MN, USA) according to the manufacturer’s recommendation. The reaction was developed with TMB and measured at 450 nm using an iEMS MF microphotometer (Thermo Labsystem, Beverly, MA, USA).

### 4.8. IL-10 ELISA

Supernatant from anti-CD180 stimulated for 24 h and unstimulated B cells were collected (SSc *n* = 5, HC *n* = 3), centrifuged and stored at −80 °C until being measured. IL-10 production was quantified using Human IL-10 DuoSet ELISA kit (Bio-Techne, Minneapolis, MN, USA) according to the manufacturer’s protocol. The reaction was developed with TMB and measured at 450 nm using an iEMS MF microphotometer (Thermo Labsystem, Beverly, MA, USA).

### 4.9. Statistical Analysis

Statistical evaluation was performed with SPSS v.25.0 statistics package (IBM, Armonk, NY, USA) using Student *t*-tests and ANOVA with post-hoc Tukey test where *p* values < 0.05 were considered significant.

## Figures and Tables

**Table 1:** Patients’ characteristics.

Characteristics	dcSSc Patients (*n* = 21)
Age (years), mean (SD)	46.43 (17.2)
Gender (female), *n* (%)	15 (71.4%)
Disease duration ^1^ (years), mean (SD)	2.05 (1.2)
Organ involvement	
MRSS mean (SD)	14.65 (7.6)
Lung fibrosis ^2^, *n* (%)	7/21 (33.3%)
Pulmonary arterial hypertension (PAH) ^3^, *n* (%)	0/21 (0%)
Renal involvement ^4^, *n* (%)	0/21 (0%)
Gastrointestinal involvement ^5^, *n* (%)	12/21 (57.1%)
Cardiac involvement ^6^, *n* (%)	9/21 (42.9%)
Current digital ulcers, *n* (%)	3/21 (14.3%)
Digital ulcers in case history, *n* (%)	5/21 (23.8%)
Digital pitting scar, *n* (%)	6/21 (28.6%)
Small joint contractures, *n* (%)	11/21 (52.4%)
Large joint contractures, *n* (%)	7/21 (33.3%)
Any joint contracture, *n* (%)	12/21 (57.1%)
Tendon friction rubs in case history, *n* (%)	1/21 (4.8%)
Arthritis, *n* (%)	2/21 (9.5%)
Subcutaneous calcinosis, *n* (%)	3/21 (14.3%)
Antibodies	
Anti-Scl-70+, *n* (%)	7/21 (33,3%)
Anti-RNA-polymerase III+, *n* (%)	3/21 (14.3%)
anti-centromere+, *n* (%)	1/21 (4.8%)
anti-PmScl+, *n* (%)	2/21 (9.5%)
anti-Th/To+, *n* (%)	1/21 (4.8%)
anti-fibrillarin+, *n* (%)	1/21 (4.8%)
Immunosuppressive therapy ^7^, *n* (%)	7/21 (33.3%)

^1^ Onset of the disease was defined as the date of the first non-Raynaud’s symptom; ^2^ pulmonary fibrosis was characterized by detection of fibrosis with high resolution CT and/or decreased forced vital capacity (FVC < 80%); ^3^ no signs suggestive of PAH on echocardiography and spirometry with diffusing capacity for carbon monoxide (DLCO) determination, therefore no right heart catheterization performed in patients; ^4^ scleroderma renal crisis was recorded as kidney involvement; ^5^ gastroesophageal involvement was established with barium swallow or esophago-gastroscopy; ^6^ cardiac involvement was defined by diastolic dysfunction or decreased left ventricular ejection fraction; ^7^ mycophenolate mofetil, azathioprine or methotrexate.

## Data Availability

Data are contained within the article.
